# Make a Brick

**Published:** 1859-02

**Authors:** 


### MAKE A BRICK.

In a late New York Observer we read "Do not conclude the Lord is not with you because things go very contrary, and he does not appear for you; he was in the ship notwithstanding the storm."

In all that Scott or Dickens ever wrote, there is not found a single sentence so fraught with solid comfort, bringing consolation so ineffably sweet to the heart all oppressed with harrowing trouble or torn asunder with saddest trials. Such a sentiment and such a sentence can never die, and will continue for ages to come to soothe the sorrowing children of humanity. And for that single sentence, we consider its unknowu author a greater benefactor to his kind than both the men whose names are written above. When Scott and Dickens have been once read they are laid away; we instinctively withdraw from a second perusal, because nothing new is expected; but the lines we have quoted will give fresh comfort to every meditative heart at every new trial, making it feel—"There is no sorrow that Heaven cannot cure."

In the "Presbyter" of Cincinnati, another excellent family paper, we read not long ago,—"The danger, temptation, and sin of the age, is the thoughtless haste to secure the world that now is, forgetful of the better, wider, everlasting world to come."

Composing a sentence like either of the two we have quoted, or doing a good deed in helping the helpless, in raising the fallen, in cheering those who are striving in privation and hard toil for an honest life, is to "make a brick" for the great building which is to pass the fiery ordeal of the general judgment, and which cannot be consumed like the "wood, and hay, and stubble," of which the scriptures have spoken.

Or, to change the simile, and bring it near a medical sense, the deeds above, and others like them, are "cordials" prepared before hand, which impart a life giving influence to those who have a right to use them in hours of trial and sickness, on a dying bed and at the judgment day!

How many of our readers have been making it a point to prepare a good supply of these "cordials" in case of emergency, when something will be needed beyond the common order of things, not the jams and jellies of the ordinary table, but the sweet-meats of the soul, of good deeds done humbly in unselfishness?

We do not know when we were more impressed with commiseration, than when reading of a great reformer, so called, dying at the age of almost ninety years, the hero of Lanark, of communism. The absorbing desire of his heart, the thing which waked up for an instant his expiring energies, the one all pervading longing of his soul was—to reach his childhood's home and there die! What feeding on dry fence rails, on the veriest husks and chaff is this. Were there no sweet memories of unselfish deeds done in the long pilgrimage of Robert Owen, upon which the soul could linger, while in another sense they could be accounted as "nothing!" The Christian has died before now in raptures ineffable, in a parched desert, on a rock of the sea, aye on the wheel and at the stake, leaning his head on the bosom of the Saviour, and breathing his life out sweetly there, panting all the while to be in heaven, in the consciousness of having endeavored, now and then at least, and O how feebly, to live for man and God, to do something to happify a brother pilgrim and help him onward to the skies.

Reader! How many "bricks" made you for 1858; what of "cordials" did you prepare in that long year of blessings, the bricks and the cordials of good deeds done for your fellow man, to the end of glorifying his Maker? How many do you purpose making the present year, for it may be your last on earth? and to lay on a bed of pain and weary suffering, to encounter the mortal agony, and have no cordial by your side to carry you through it all, happily, triumphantly, how dreadful!—Go this minute and do some good deed to somebody, for you may die to-morrow, and if you do not die to-morrow, "repeat the prescription" every day until you do.

